# Using reinforcement algorithms to improve the collaboration efficiency of entrepreneurial teams

**DOI:** 10.1371/journal.pone.0343247

**Published:** 2026-03-11

**Authors:** Jieqiong Wang, Linghong Jiang

**Affiliations:** 1 School of Business, Zhengzhou University of Aeronautics, Zhengzhou, Henan, China; 2 Jiangxi Health Vocational College, Nanchang, China; Grigore T Popa University of Medicine and Pharmacy Iasi: Universitatea de Medicina si Farmacie Grigore T Popa lasi, ROMANIA

## Abstract

Entrepreneurial Team (ET) plays an essential role in the business process by driving innovation and optimizing ideas via adaptability, collaboration, and resourcefulness. The team performance is continuously affected because of resource imbalance, poor communication and inefficient task allocation. The importance of ET in organization growth is the main reason for this analysis. Therefore, this work uses Multi-Agent Reinforcement Learning (MARL) to handle efficient dynamic decisions and coordination to improve ET efficiency in dynamic and complex environments. The main intention of this work is to improve resource utilization, communication efficiency and optimize task allocation. During the analysis, Proximal Policy Optimization (PPO) is utilized to direct agents toward achieving collaborative goals. In every state, the agent receives rewards and penalties for their actions, which helps meet the organization’s goal with minimum time and improves the overall task completion rate. This process is evaluated using different case studies like software development, optimized manufacturing and logistic coordination, which helps to validate the system’s adaptability in various scenarios. In addition, different hypotheses are validated via case studies and metrics such as defect resolution, collaboration quality, operational efficiency, resource optimization, and task completion rate. Thus, the work highlights the impact of MARL in ET to ensure the highest performance in a dynamic environment.

## 1. Introduction

The ET characteristics, significance, and dynamics are utilized to understand every enterprise and improve its performance to meet the organization’s goal. The ET consists of individuals sharing their knowledge, responsibilities, rewards, and risks to develop and maintain the new venture [1–2]. The entrepreneurial team members are interdependent; they work to meet the mutual goal, influencing the business’s performance and decision-making efficiency [3]. The founding teams of entrepreneurs often recognize the success of entrepreneurial ventures. Several studies [4] reveal that teams leading a venture are more viable and profitable and have more growth potential than those an entrepreneur started. Since entrepreneurial skills, experiences, and views are needed to handle the challenges and risks of establishing a new firm, entrepreneurial teams are better equipped to provide such skills than individuals [5]. However, the ET faces several challenges, such as uncertainty, high autonomy, resource constraints and dynamic environment. The ET process and functions operate under high autonomy conditions, creating ambiguity [6] while differentiating the agent’s role and responsibilities. The ET ambiguity causes team conflicts while analyzing and allocating tasks to the team members. Most of the ETs developed with limited resources, affecting team performance due to the strained relationships between the members [7,8]. Finally, the ET environment is dynamic, in which the requirements and business objectives change frequently; therefore, the team needs to adapt to the changing environment quickly. These challenges are addressed by several strategies [9], such as selecting the right team members, creating a shared and clear vision and defining exact roles and responsibilities. These strategies are used to understand the team member’s skills and values, which helps to learn the team objectives. According to their understanding, roles and responsibilities are given to the team to make effective decisions in a dynamic environment [10]. Even these strategies face conflicts, disagreement, and resource scarcity issues due to improper communication efficiency, which affects the entire task allocation and completion rate. In addition, entrepreneurial ventures utilize rule-based [11] and static frameworks [12–13] to improve ET performance. Still, they face difficulties differentiating the environmental conditions and team priorities.

The Research Questions in this study are:

- How might a Multi-Agent Reinforcement Learning (MARL) framework improve goal alignment, communication, and task allocation in entrepreneurial teams?
- How well can MARL-based solutions balance workload distribution, maximise resource productivity, and minimise operational downtime in entrepreneurial teams?
- How can MARL incorporate adaptive learning processes to help entrepreneurial teams adjust to dynamic priority shifts and make better decisions?

Therefore, this work uses Multi-agent reinforcement learning (MARL) to improve the adaptability and collaboration between the members and ensure effective interaction in the dynamic ET environment. This framework observes the environment and permits agents to decide depending on the experience and observation that maximize the ET collaborative efficiency. Multi-Agent Reinforcement Learning (MARL) can represent and enhance collaboration efficiency between several agents. A paradigm is built that integrates Proximal Policy Optimization (PPO) with customized reward systems to optimize entrepreneurial teams’ performance regarding workloads, conflicts, and productivity. The effective utilization of rewards and penalty schemes in the ET process helps improve overall collaboration and reduce delays while understanding the task in a dynamic environment. This process is developed in different agent environments, and the efficiency of the MARL is validated using a set of hypotheses and case studies. The case studies are created in the product lifecycle, manufacturing and logistic coordination, which helps to validate the ET’s adaptability, task allocation rate, scalability, downtime duration and operational efficiency. Then the overall contribution of this work is listed below.

- To analyze the ET dynamic environment using the MARL approach to enhance team collaboration by optimizing goal alignment, communication and task allocation.
- To maximize resource utilization by balancing the workload, resource productivity and minimizing the downtime according to the ET environment.
- To enable the ET to adapt to the priority change by incorporating adaptive learning to improve decision-making efficiency.

This manuscript is organized as follows: Section 2 deliberates the comparable study of ET from different researcher’s perspectives. Section 3 describes the working process of MARL-based ET collaboration efficiency analysis. Section 4 explains the various hypotheses and case studies to validate the MARL systems, and the conclusion is described in Section 5.

## 2. Comparable research analysis

Krawczyk-Bryłka B. et al. [14] analyze the collaboration principles importance in entrepreneurial teams for improving member satisfaction. The ETICP tool is utilized to identify the difference between established and nascent teams according to their relationship, which is directly linked with the venture performance. The author uses a set of questionnaires with 6-item scale values, and nine collaborative principles are utilized to evaluate the entrepreneurial team member’s satisfaction. This study does well on entrepreneurial team building, however it doesn’t analyze Poland’s leading research and education programs for entrepreneurs. Han et.al [15] advocated selecting entrepreneurial team members using a statistical LSTM chain network and social network analysis (SLSTM-CAN), a novel approach. This work intends to choose the high potential members for the team to improve the start-up success rate. The selection procedure is performed to explore the social network structure, collaborative capacities, and connections. This information is processed with the help of an introduced classifier that permits a forecasting system to identify the entrepreneurial members according to the dynamics and trends. The classifier-based member selection improves business growth by up to 25% and 30% in adaptability.

Chughtai M. S. et al. [16] introduced adaptive learning to improve organizational innovations by understanding leadership roles. This work is implemented using SPSS, Smart-PLS and AMOS software, and a simple random sampling approach is utilized for data collection. The gathered data is explored with the help of the interaction-effect analysis approach that is used to validate the correlation and reliability of the team. Along with this, leadership ability, role and self-efficacy are evaluated to improve the innovations in the organization. Therefore, the study focuses on leaders’ self-efficacy to enhance organizational innovations. Bouncken R. et al. [17] analyzed the fostering of entrepreneurship coworking space to improve the sharing and digital economy. This study provides the guidelines for understanding the dynamic condition of entrepreneurship coworking space to offer collaborative knowledge for sharing the resources to improve business growth. This study enables the interaction between the team members to manage flexibility and adaptability while driving innovations. Thus, the system provides the platform for improving the economic landscape and growth and effectively gives collaboration opportunities.

Covin, J. G. et al. [18] investigate the constructs of Team Entrepreneurial Orientation (TEO) and Individual Entrepreneurial Orientation (IEO), portraying a theory of how both orientations are likely to affect performance at the organizational level. The authors constructed and justified the IEO measurement scale and emphasized its essential components: risk, proactiveness, and innovativeness. They suggest that TEO is formed when team members have different goals but create together, and their goals are the same, facilitating effective team processes and performance. The study uses qualitative comparative analysis to identify successful configurations in teams, emphasizing the individual and the team entrepreneurs. Donbesuur F. et al. [19] inspect the correlation between the new venture performance according to the entrepreneurial orientations by identifying their actions and contingent roles. The orientation observes the dynamic environment based on the actions, business networking, opportunity discovery and support systems. The observed environment details help to provide robust collaboration, improving the overall performance of new ventures. Sutrisno, S., et al. [20] considered the contribution of information technologies for introducing new ideas and growth of business structures in the founding. The research shows that entrepreneurs are required to make good use of techniques to improve the creation of products, better the organization of processes, and reach more markets. The authors show that information technology accelerates business innovation and improves customer experience, making it essential for corporate growth by using qualitative analysis and secondary data collection. The results portray the need for entrepreneurs to embrace technology if they are to stay competitive in the business environment. On the other hand, Du et al. [21] proposes a MARL framework that is both scalable and safety-constrained for complex multi-agent systems. Enhanced coordination in the face of uncertainty can be achieved through the integration of robust policy learning and adaptive risk management. When it comes to dynamic situations, such as entrepreneurial teams, where agent collaboration and safety are essential for decision-making, the study is extremely significant. In their study [22], Jeloka and colleagues create a MARL technique that makes use of mean-field interactions to replicate the behaviour of large-scale, competitive teams. Providing significant parallels to the optimisation of collaborative tactics within big entrepreneurial teams that are navigating dynamic and competitive business situations, the research clearly demonstrates how PPO can scale to a large number of agents while retaining learning stability. A unique form of PPO called PPO-ACT is presented by Yang et al. [23]. This variant makes use of adversarial curriculum transfer in order to increase cooperation in spatial public goods games. Learning is dynamically adapted through this approach, which makes it suited for contexts that involve complicated and ever-changing teams. The lessons it teaches are valuable to entrepreneurial teams who are looking to improve their agility and their capacity to efficiently coordinate tasks. From the various researchers’ opinions, the information technology framework, data analysis, and innovation exploration process improve ET efficiency. However, the ET faces difficulties because of improper communication and resource allocation, which affects the entire task allocation and completion rates. These difficulties completely affect the efficiency of ET collaboration. Therefore, the research difficulties are addressed by including reinforcement learning to improve the ET collaboration efficiency.

Recent Research on AI-Based Collaborative Systems and Reinforcement Learning in Entrepreneurial or Multi-Agent Contexts as shown in Table 1 below:

**Table 1:** Comparative analysis of recent research on AI-based collaborative systems and reinforcement learning in entrepreneurial or multi-agent contexts.

Authors	Objective	Methods	Findings	Advantages	Disadvantages	Research Gap
Zheng [24]	To design a learning platform with personalized entrepreneurial project recommendations for college students	Group recommendation algorithm integrated with learning behavior analysis	Increased student engagement and personalized learning pathways	Tailors suggestions based on group interests and behavior patterns	Limited adaptability to dynamic student interest shifts	Need for real-time learning adjustment and continuous feedback mechanisms
Lv et al. [25]	Optimize team formation in large organizations using AI	Deep Reinforcement Learning (DRL) for adaptive team formation	DRL formed more balanced, skill-diverse teams improving team performance	Dynamic adaptability to organizational structures and individual skills	Requires large training data and fine-tuning of reward systems	Integration with real-world organizational dynamics remains unexplored
Duraimutharasan et al. [26]	Enhance efficiency and decision-making in control engineering through human-AI collaboration	Hybrid control systems with AI-assisted decision models	AI support led to significant efficiency and responsiveness gains in industrial control	Combines human expertise with intelligent automation	Potential issues with human trust and AI transparency	Lacks domain-specific performance benchmarks and validation in high-risk systems
Zhou et al. [27]	Optimize task scheduling in multirobot systems using MARL and graph convolution	Multi-Agent RL + Heuristic Graph Convolution for service-aware scheduling	Improved scheduling accuracy, service balance, and execution efficiency	Joint learning of task priority and service capacity improves outcomes	Complexity of coordination in large robot fleets	Limited exploration in human-robot collaborative environments
Tu et al. [28]	Improve collaboration between UAVs and vehicles in mobile crowdsensing	Evolutionary Multi-Agent RL with adaptive role learning	Achieved high adaptability and coverage in sparse dynamic environments	Learns and adjusts roles dynamically for collaboration in real-time	Needs high compute for training; less suitable for constrained devices	Future scope in energy-aware and privacy-preserving multi-agent collaboration
Zheng et.al	To design a learning platform with personalized entrepreneurial project recommendations for college students	Group recommendation algorithm integrated with learning behavior analysis	Increased student engagement and personalized learning pathways	Tailors suggestions based on group interests and behavior patterns	Limited adaptability to dynamic student interest shifts	Need for real-time learning adjustment and continuous feedback mechanisms
Lv et al.	Optimize team formation in large organizations using AI	Deep Reinforcement Learning (DRL) for adaptive team formation	DRL formed more balanced, skill-diverse teams improving team performance	Dynamic adaptability to organizational structures and individual skills	Requires large training data and fine-tuning of reward systems	Integration with real-world organizational dynamics remains unexplored
Duraimutharasan et al. [29]	Enhance efficiency and decision-making in control engineering through human-AI collaboration	Hybrid control systems with AI-assisted decision models	AI support led to significant efficiency and responsiveness gains in industrial control	Combines human expertise with intelligent automation	Potential issues with human trust and AI transparency	Lacks domain-specific performance benchmarks and validation in high-risk systems
Zhou et al.	Optimize task scheduling in multirobot systems using MARL and graph convolution	Multi-Agent RL + Heuristic Graph Convolution for service-aware scheduling	Improved scheduling accuracy, service balance, and execution efficiency	Joint learning of task priority and service capacity improves outcomes	Complexity of coordination in large robot fleets	Limited exploration in human-robot collaborative environments
Tu et al.	Improve collaboration between UAVs and vehicles in mobile crowdsensing	Evolutionary Multi-Agent RL with adaptive role learning	Achieved high adaptability and coverage in sparse dynamic environments	Learns and adjusts roles dynamically for collaboration in real-time	Needs high compute for training; less suitable for constrained devices	Future scope in energy-aware and privacy-preserving multi-agent collaboration

## 3. Context of Reinforcement Learning (RL) to team collaboration in entrepreneurial team (ET)

### 3.1 Impact of RL framework

The main objective of this framework is to enhance the collaboration and team function in entrepreneurial teams. The work uses Reinforcement Learning (RL) to optimize decision-making, communication, conflict resolution, and task allocation in a dynamic environment. The manual task allocation in ET causes overburdened and inefficient problems that reduce the entire ET efficiency. The RL algorithm uses the agent and action concept to address task allocation issues by considering every member as the agent. For every agent’s workload, task priorities, skill, and balancing criteria help choose the team member to assign the specific task. Then, the team member’s improper communication creates misunderstanding issues, affecting decision-making efficiency. The introduced RL approach observes the interaction patterns, which helps identify the exact points and eliminate irrelevant interactions, improving ET clarity and decision-making efficiency. The ET team faces conflicts because of resource constraints, whereas the RL predicts the previous conflict patterns and provides solutions that reduce the delay and improve satisfaction. The ET functions in a dynamic and uncertain environment, so collaboration fails. The incorporated RL techniques provide effective, scalable, and reliable strategies for improving overall collaboration while making decisions. During the analysis, RL observes the agent’s actions that enhance ET’s cohesion and productivity. Then, the objective of this work is defined in Equation (5), which explains the collaborative efficiency (F). These objectives are created with certain constraints, which are described as follows.

#### Unified constraints.

As discussed, the RL is utilized in several parts of ET, such as task allocation, decision-making, etc. For a particular task i, the team member $M_{j}$ is assigned depending on the priority $p_{i}\;and\;$their skill set $S_{j,k\;}$, workload $W_{j}$. Then reward $R_{i,j}$ should be allocated to the $M_{j}$ depending on the performance (pf). Then, the task allocation is defined as $\sum_{i=\text{1}}^{N}\sum_{j=\text{1}}^{M}R_{i,j}.A_{i,j}$ with specific constraints as defined in Equation (1)


$$\;\begin{array}{c} \;\begin{array}{c} A_{i,j}\in (\text{0},\text{1})\\ \sum_{j=\text{1}}^{M}A_{i,j}=\text{1} \end{array}\}task\;assigned\;to\;M_{j}\\ \;\begin{array}{c} W_{j}+p_{i}\leq W_{max}\\ S_{j,k}\geq S_{i} \end{array}\}\;workload\;and\;skill\;\\ \;constraint \end{array}\}$$ (1)

Once the task is assigned, the pfis improved with the help of proper communication $C_{i,j},$ relevant communication $R_{c}$, time $T_{c}\;$spent on$C_{i,j}$, task urgency $U_{i}$ and $C_{i,j}$ quality feedback $F_{c}$. Then the $C_{i,j}$ efficiency is measured with the specific constraint that is defined in Equation (2)


$$\;\begin{array}{c} \text{max}\sum_{i=\text{1}}^{N}\sum_{j=\text{1}}^{M}\left(R_{c}.F_{c}\right)-\alpha \sum_{i=\text{1}}^{N}T_{c}\\ \;\begin{array}{c} T_{c}\leq \;T_{max}\\ R_{c}\geq \delta \end{array}\}\;Time\;and\;relevance\;\\ \;constraints \end{array}\;\;\;\}$$ (2)

During the $C_{i,j}$, the task conflicts (k) occur, which is minimized with the help of the resolution priority $\left(P_{k}\right)$, resource allocation $\left(R_{k}\right)$ and dissatisfaction score $\left(D_{k}\right)$. Therefore, the conflicts are optimized with the specified constraint that is defined in Equation (3)


$$\;\begin{array}{c} -\beta \sum_{k=\text{1}}^{C}D_{k}+\gamma \sum_{k=\text{1}}^{C}R_{k}\\ \;\begin{array}{c} \sum_{j=\text{1}}^{M}R_{j,k}\leq R_{max}\\ P_{k}\geq \tau \end{array}\}\;resource\;and\;priority\;\\ \;constraints \end{array}\}$$ (3)

After reducing the conflicts k environment $\varepsilon_{t}$ should be observed, and adjust the ET strategies $\theta_{t}$ for minimizing the loss function $L\left(\theta_{t},\varepsilon_{t}\right).$ Then, the learning process for task i is defined with constraint in Equation (4)


$$\;\begin{array}{c} \underset{\theta_{t}}{\text{min}}E\left(L\left(\theta_{t},\varepsilon_{t}\right)\right)\\ \theta_{t+\text{1}}=\theta_{t}-\eta \nabla \theta_{t}\;L\left(\theta_{t},\varepsilon_{t}\right) \end{array}\}$$ (4)

These overall constraints and descriptions are used to improve the $M_{j}$ productivity $P_{j}$, ET goal score G and cohesion score $C_{j}$. Then, the ET productivity is defined as $\text{max}\sum_{j=\text{1}}^{M}\left(P_{j}+\lambda C_{j}\right)+\mu G$. Finally, the overall objective of this work is defined in Equation (5) based on the above computation constraints.


$$F=\text{max}[\sum_{i=\text{1}}^{N}\sum_{j=\text{1}}^{M}R_{i,j}.A_{i,j}+\sum_{i=\text{1}}^{N}\sum_{j=\text{1}}^{M}\left(R_{c}.F_{c}\right)-\alpha T_{c}-\beta \sum_{k=\text{1}}^{C}D_{k}+\gamma \sum_{k=\text{1}}^{C}R_{k}+\mu G+\lambda \sum_{j=\text{1}}^{M}C_{j}+\nu +\sum_{j=\text{1}}^{M}P_{j}\;\backslash \;(\text{5})$$ (5)

### 3.2 Research methodology

**Fig 1:**
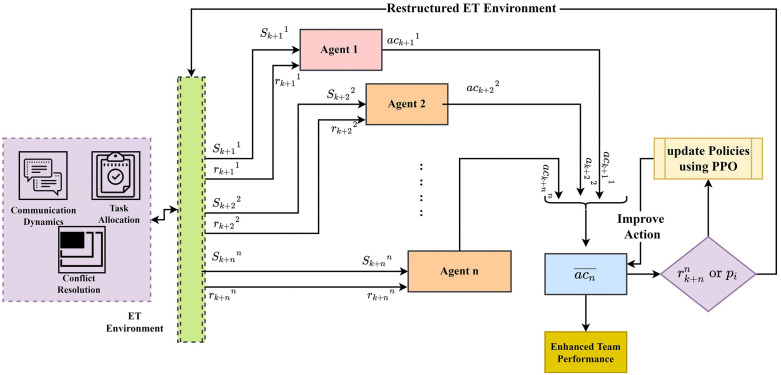
Framework of MARL in ET.

The entrepreneurial team uses a Multi-Agent Reinforcement Learning (MARL) framework to ensure ET collaborations and improve team efficiency in a dynamic environment. Initially, the environment was designed considering several components, such as conflict resolution, communication dynamics, and task allocation. These components cover task priorities, cohesion, productivity, and workload. These variables changed depending on the external events and agent actions. In the ET design, each team member is considered an agent with specific skills and decision-making capabilities, which helps improve the team’s performance. Every agent $\left(a_{j}\right)$ is involved in the ET components to meet their team goal and objectives. According to the $a_{j}^{\prime }s$ actions, rewards are allocated, and penalties are provided for uncertain tasks like imbalance workload distribution and communication delay. This process improves the $a_{j}$ actions to reach the team goal effectively. This process is continuously performed and is measured in terms of a continuous loop, and with every iteration, $a_{j}^{\prime }s$ actions are observed based on the policies. The ET environment observes these actions and provides feedback in terms of penalties $\left(p_{i}\right)$ or rewards $\left(r_{i}\right)$. The utilized policies are frequently updated using the proximal policy optimization (PPO) algorithm in which $a_{j}$ continuously update their decisions. The PPO algorithm is an efficient and stable approach that optimizes the $\left(a_{j}\right)$ performance. This process is repeated, and continuous output is obtained, which enhances the team performance computed in terms of communication relevance conflict and improves the team’s productivity. The structure of MARL is illustrated in Fig 1.

Fig 1 illustrates the framework of MARL-based collaboration efficiency in the ET environment. The ET environment has a few design components, such as communication flow, task allocation, and goal setting, which help to initiate the $M_{j}$ interactions for the particular task $t_{i}$ to meet the business objectives. Then, the ET state is defined by observing the $T_{i}$ concerning their skill requirement, priorities, workload ($W_{j})$ of $a_{j}$, communication between the agents $\left(C_{i,j}\right)$, resource allocation conflicts $\left(R_{k}\right)$, team goal score (G), productivity score $\left(P_{j}\right)$, cohesion score $\left(C_{j}\right)$ and dissatisfaction score $\left(D_{k}\right)$. According to these parameters, the $ET_{S}$ is defined for agent action $ac_{t}$ is defined using Equation (6)


$$\;\begin{array}{c} S_{t+\text{1}}=f\left(S_{t},\;ac_{t}\right)\\ S_{t}=\left\{T_{i},\;C_{i,j},W_{j},\;R_{k},\;G,\;P_{j},C_{j}\;and\;D_{k}\right\} \end{array}\}$$ (6)

After defining the $ET_{S}$, agent $a_{j}$ has to be defined to perform the particular task $T_{j}$. The $a_{j}$ is defined by the respective skill set $\left(S_{j,k}\right),\;W_{j}\;and\;\pi_{j}$ (decision policies). The $a_{j}$ intends to enhance the $r_{j}$ by performing task allocation $\left(A_{i,j}\right)$ communication $\left(C_{i,j\;}\right)$ and conflict resolve $\left(R_{k}\right)$. The rewards are allocated to the $a_{j}$ based on the effective task completion ($R_{t})$, communication ($R_{c})$, conflict resolution ($R_{cf})$ and team workload balance ($R_{te})$. Then the $r_{j}$ is estimated using Equation (7)


$$\;\begin{array}{c} r_{j}=R_{t}+R_{c}-\beta R_{cf}+R_{te}\\ R_{t}=\sum_{i=\text{1}}^{N}\sum_{j=\text{1}}^{M}r_{i,j}.A_{i,j}\\ R_{c}=\sum_{i=\text{1}}^{N}\sum_{j=\text{1}}^{M}\left(R_{c}.F_{c}\right)-\alpha T_{c}\\ R_{cf}=\sum_{k=\text{1}}^{C}D_{k}+\gamma \sum_{k=\text{1}}^{C}R_{k}\\ R_{te}=\mu G+\lambda \sum_{j=\text{1}}^{M}C_{j}+\nu \sum_{j=\text{1}}^{M}P_{j} \end{array}\}$$ (7)

In Equation (7), $r_{j}$ is estimated from the successful task assignments $R_{t}$, relevant communication $R_{c}$, unresolved conflict penalty $R_{cf}$ and team productivity-cohesion $R_{te}$. After providing the $r_{j}$ to $a_{j}$ the policies need to be updated for $a_{j}$ learn the policies to improve the rewards for attaining efficiency and stability. The policies are updated with the help of proximal policy optimization (PPO), which helps manage ET stability, efficiency, and scalability. The PPO-based policy update procedure enhances the environment interactions and ability to handle the complex environment. The PPO upgrade the $\pi_{\theta }$ policies by exploitation and exploration during the policy update, and the updated values are close to the ${\pi \theta }_{old}$ previous policies. Therefore, the main objective of the PPO-based policy update is defined in Equation (8)


$$\;\begin{array}{c} L_{total}(\theta )=L(\theta )-C_{\text{1}}L_{V}(\theta )+c_{\text{2}}L_{E}(\theta )\mathrm{\backslash vspace}1mm\\ L(\theta )=E_{t}\left[\text{min}\left(r_{t}(\theta )\hat{{AC}_{t}},clip\left(r_{t}(\theta ),\text{1}-\varepsilon ,\text{1}-\epsilon \right)\hat{{AC}_{t}}\right)\right]\mathrm{\backslash vspace}1mm\\ L_{E}(\theta )=-E_{t}\left[\pi_{\theta }\left({ac}_{t}|s_{t}\right)\text{log}\pi_{\theta }\left({ac}_{t}|s_{t}\right)\right]\mathrm{\backslash vspace}1mm\\ L_{V}(\theta )=\frac{\text{1}}{\text{2}}E_{t}\left[{\left(V_{\theta }\left(s_{t}\right)-G_{t}\right)}^{\text{2}}\right] \end{array}\}$$ (8)

**Fig 2:**
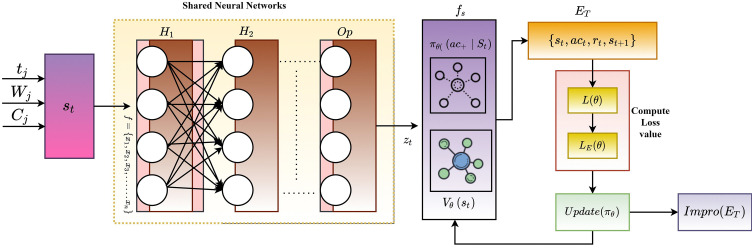
Structure of PPO in ET.

The PPO objective $L_{total}(\theta )$ is achieved from various key components such as surrogate objectives (L(θ)), value function $L_{V}(\theta )$ and entropy bonus $L_{E}(\theta )$. The $L_{E}(\theta )$ used to improve the exploration process while examining the ${AC}_{t}$ on specific state $s_{t}$. In addition, the PPO trains the $V_{\theta }\left(s_{t}\right)$ value function to predict the return with the help of the cumulative reward value $G_{t}$. As discussed, the policies $\pi_{\theta }$ updated depending on the previous policy information; therefore, the rate value is estimated as $r_{t}(\theta )=\frac{\pi_{\theta \left({ac}_{t}|s_{t}\right)}\;}{{\pi \theta }_{old}\left({ac}_{t}|s_{t}\right)}$. During the computation, clipping factor ϵ is used to manage the ET stability and weight values $c_{\text{1}}\;and\;c_{\text{2}}$ are utilized while computing the PPO objectives. Then, the overall structure of PPO based policy update process is shown in Fig 2.

Fig 2 illustrates the PPO structure in ET to improve the collaboration between the team members to complete the task. The input layer gets the $S_{t}$ inputs ($t_{j},\;W_{j}\;and\;C_{j})$ as input, the shared neural network processes extract the features for value and policy functions. The main intention of the network is to get features like task progress, communication dynamics, and workload distribution for particular tasks. During the analysis, convolution layers are utilized to derive the high-level features for input $S_{t}$. The extracted $f_{s}$ reduces the overhead and redundancy issues while analyzing the $E_{T}$ environment. The derived features and patterns are denoted as $z_{t}$ which used to get the $\pi_{\theta \left({ac}_{t}|s_{t}\right)}$ and $V_{\theta }\left(s_{t}\right)$ values from policy and value function networks. Theinput layer receives the $S_{t}$ information, which is defined as $S_{t}=\left(x_{\text{1}},x_{\text{2}},\ldots .x_{n}\right)$ that covers continuous and discrete values that need to be normalized for further computation. The $S_{t}$ is fed into the hidden layer that uses the $w^{l}\;and\;b^{l}$ for computing the output value. Then, the hidden layer $h^{l}$ computation is defined in Equation (9)


$$\;\begin{array}{c} h^{\left(\text{1}\right)}=\phi \left(W^{\left(\text{1}\right)}s_{t}+b^{\left(\text{1}\right)}\right)\\ h^{\left(\text{2}\right)}=\phi \left(W^{\left(\text{2}\right)}s_{t}+b^{\left(\text{2}\right)}\right)\\ \vdots \\ \vdots \\ h^{\left(k\right)}=\phi \left(W^{\left(k\right)}h^{\left(k-\text{1}\right)}+b^{\left(k\right)}\right)\; \end{array}\;\;\;\;\}$$ (9)

In Equation (9), first hidden layer output is defined as $h^{\left(\text{1}\right)}$ that is computed by utilizing ϕ(.)=max(0,x) activation function that is computed as $\phi (x)=\frac{\text{exp}(x)-\text{exp}(-x)}{\text{exp}(x)+\text{exp}(-x)}$. Similarly, the k hidden layers compute the output for deriving the information for policy and value functions. The hidden layer is selected depending on the $s_{t}$ space, which is used to identify the relationship between the features. The output is computed as $z_{t}=h^{\left(L\right)}$ which is compressed depending on the $s_{t}$. The estimated $z_{t}$ value is divided into the policy and value functions to improve the overall ET performance. The policy network estimates the action probabilities with the help of the SoftMax activation function, which is calculated using Equation (10), and the value network identifies the state value with the help of the linear layer.


$$\;\begin{array}{c} \pi_{\theta \left({ac}_{t}|s_{t}\right)}=\frac{\text{exp}\left(u_{a}\right)}{\sum_{b\in A}\text{exp}\left(u_{b}\right)}\\ u_{a}=W^{policy}z_{t}+b^{policy}\\ \;V_{\theta }\left(s_{t}\right)=W^{value}z_{t}+b^{value} \end{array}\;\;\}$$ (10)

After computing the $\pi_{\theta \left({ac}_{t}|s_{t}\right)}$ and $V_{\theta }\left(s_{t}\right)$ which network gradient value is estimated from the computation for deriving the $L_{policy}$ is estimated from Equation (8) $E_{t}\left[\text{min}\left(r_{t}(\theta widehat{AC}_{t},clip\left(r_{t}(\theta ),\text{1}-\varepsilon ,\text{1}-\epsilon \right)\hat{{AC}_{t}}\right)\right]$. The $L_{policy}$ value is computed with the help of an advantage estimate $\hat{{AC}_{t}}$ and probability ratio $r_{t}(\theta )$. In addition, $L_{value}$ is computed for $V_{\theta }\left(s_{t}\right)$ which is used to estimate the deviation between the actual and predicted return value that is derived as $\frac{\text{1}}{\text{2}}E_{t}\left[{\left(V_{\theta }\left(s_{t}\right)-G_{t}\right)}^{\text{2}}\right]$. During the loss computation, the exploration is encouraged by computing the $L_{E}$ value, and the total loss is estimated as $L_{total}(\theta )=L(\theta )-C_{\text{1}}L_{V}(\theta )+c_{\text{2}}L_{E}(\theta )$. According to the computed $L_{total}(\theta )$, environment interactions are improved by considering their $s_{t},ac_{t}\;and\;r_{t}.$ Then, the policies are updated frequently to improve the overall ET environment. The combined neural network is a single device for embedding the policy and the value functions. It provides efficient computations, uniformity of representation, and quick convergence. Because of this, the network eliminates unnecessary computing by sharing weights while maintaining specificity and generality throughout learning different tasks. Then, the overall working process of MARL pseudocode is described in Pseudocode 1 below:

### Pseudocode for MARL

**Table:** 

*Initialization:* ET, $a_{n}$, $sh_{net}$, $\pi_{\theta }$ *and* $V_{\theta }$				
$Hyper_{parameter}:\;\alpha ,\gamma ,\varepsilon ,L_{entropy},L_{V}(\theta ),\;M\;and\;T\;$				
**Define** $r_{stru}:R_{t},\;T_{c}\;and\;C_{s}$				
for e=1 to M+1				
	$state\;s\leftarrow env_{rest}()$			
	$t_{r}\leftarrow [\text{0}\;for\;in\;range\;\left(a_{n}\right)]$			
	for s=1 to T+1			
		Action A←[]		
		$for\;a\;in\;A_{n}$		
			$z\leftarrow sh_{net}.extra_{f}(s)$	
			$\pi_{\theta }\leftarrow a.poli_{net}(z)$	
			$a\leftarrow \pi_{\theta }.sample\;$()	
			A. append (a)	
		$new_{s},r,\;done\leftarrow env.s(A)$		
		for i, a in enumerate (A)		
			$a.store_{tran}(s,A[i],r[i],new_{s},\;done)$	
			$r_{total}[i]+=r[i]$	
		$s\leftarrow new_{s}$		
		If done		
			break	
	For a in A			
		tra←a.get−tra()		
		$G_{t},\;\hat{A}\leftarrow com_{retu_{adv}}(tra,\gamma ,V_{\theta })$		
		$for\;E\;in\;range(P_{E})$		
			$for\;b\;in\underset{bat}{\text{min}}\left(tran\right)$	
				$\pi_{\theta_{old}}\leftarrow a.p_{net}\left(b_{s}\right).detach()$
				$\pi_{\theta_{new}}\leftarrow a.p_{net}\left(b_{s}\right)$
				$ratio\leftarrow \pi_{\theta_{new}}/\pi_{\theta_{old}}$
				$L_{policy}\leftarrow \;-\text{min}(ratio*\hat{A},\;clip\;(ratio,\text{1}-\epsilon ,\text{1}+\epsilon *\hat{A})$
				$L_{V}(\theta )\leftarrow c_{value}*mse\left(V_{\theta }\left(b_{s}\right)\right)$
				$L_{entropy}\leftarrow c_{entropy}*Entropy\;\left(\pi_{\theta_{new}}\right)$
				$L_{total}\leftarrow L_{policy}+L_{V}-L_{entropy}$
				$a.opt.zero_{grad}()$
				$L_{total}.backward()$
				$a.opt.zero_{s}()$
	${\text{log}}_{per}\left(E,\;r_{total}\right)$			

**Fig 3:**
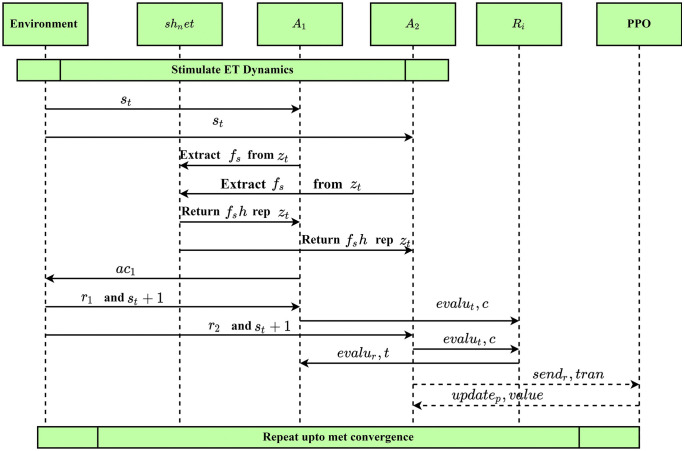
Interaction process in ET using MARL.

Pseudocode 1 illustrates for MARL-based ET performance improvement. The algorithm works started from $ET_{initi}$, $A_{initi}\;and\;r_{i}$. The selected $r_{i}$ are updated using PPO, which balances exploitation and exploration while improving ET performance. Effectively utilizing agent, reward and actions-based task analysis processes improve task allocation excellence, communication relevances, resolving conflicts, productivity and team cohesion. Then, the overall interaction of every member in ET is illustrated in Fig 3.

**Fig 4:**
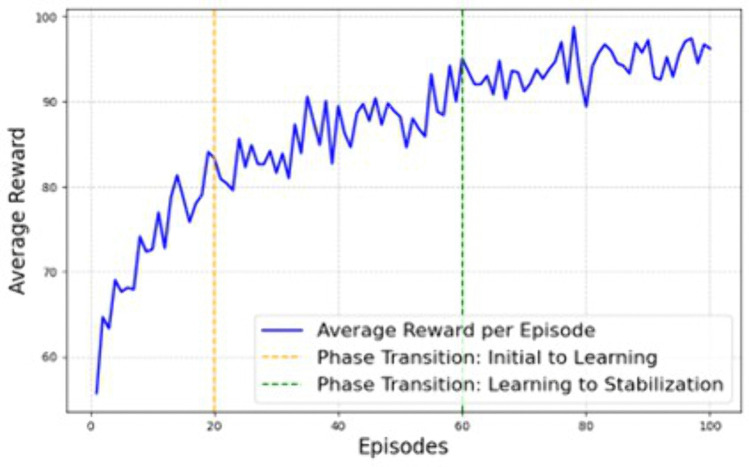
Graphical analysis of $r_{j}$.

According to the Fig 3, it clearly shows that $r_{j}$ mechanism played an essential role in improving the efficiency of ET collaboration. The $r_{j}$ the process used to regulate the $a_{j}$ behaviour towards the $t_{j}\;$ allocation and workload $W_{j}$ balance. The $r_{j}$ mechanism covers the immediate rewards and task delay rewards, which helps analyze the ET performance. Then the $r_{j}$ is computed at t as $R_{t}=\alpha \sum_{i=\text{1}}^{N}C_{i}+\beta \left(\text{1}-\frac{\text{max}\left(W_{i}\right)-\text{min}\left(W_{i}\right)}{\sum_{i}W_{i}}\right)-\gamma \sum_{j}D_{j}$. From the computation, $R_{t}$ is estimated from unsolved conflicts $D_{j}$, weights (α,β and γ), task completion score $C_{j}$ and workload $W_{i}$. Then, the reward scenario is analyzed using a 5-agent ET situation with dynamic $t_{j}$, communication delay and skill sets. During the analysis, 100 simulation cycles are utilized because of task complexities. In this scenario, the reward efficiency is explored in terms of the initial phase (0–20 episodes), learning phase (20–60 episodes) and stabilization phase (above 60 episodes). In the initial phase, $a_{j}$ prioritize the rewards according to the team dynamics. In the learning phase, $a_{j}$ adapt to the $r_{j}$ process tasks are allocated to minimize the conflicts and, finally, the optimal $r_{j}$ are allocated to the members to manage the collaboration. According to the analysis, the obtained $r_{j}$ value is described in Fig 4.

**Fig 5:**
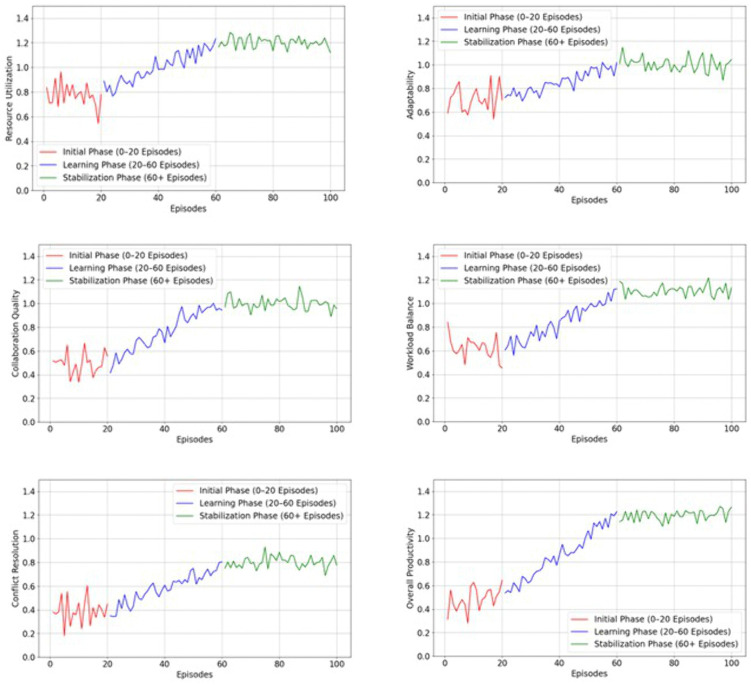
Reward mechanism efficiency analysis. **(a)** $re_{utilization}$ **analysis (b)** $R_{a}$ **Analysis (c)** $c_{q}$**analysis (d)** $w_{b}$ **analysis (e)** $R_{c}$**analysis (f)** $R_{p}$ **analysis.**

Fig 4 clearly shows that $r_{j}$ approach is effectively utilized to improve ET performance while deriving collaboration efficiency. The gradual increment of $r_{j}$ the reinforcement learning approach helps meet the team goal with minimum computation difficulties. In addition, the $r_{j}$ are provided depending on the task completion and workload balance, directly indicating team improvement and stabilization in a dynamic environment. The $r_{j}$ efficiency is further evaluated in terms of resource utilization $\left(re_{utilization}\right)$, workload balance $\left(w_{b}\right)$, collaboration quality $\left(c_{q}\right)$, adaptability $\left(R_{a}\right)$, resolution conflict $\left(R_{c}\right)$ and overall productivity $\left(R_{p}\right)$. For these metrics, the efficiency of the MARL approach on ET performance is evaluated, and the respective results are shown in Fig 5.

**Fig 6:**
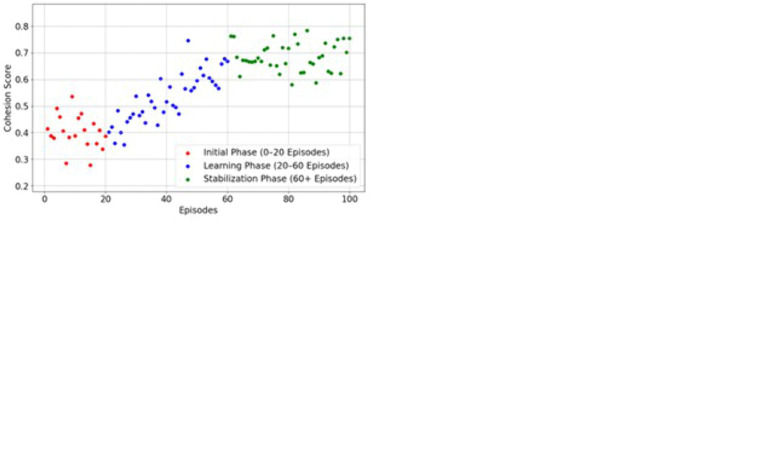
Collaboration efficiency analysis. **(a)** $tc_{time}$ **analysis (b)** $c_{effi}$ **Analysis (c)** $cf_{speed}$**analysis (d)** $tc_{score}$ **analysis.**

Fig 5 illustrates that the MARL-based optimized reward mechanism improves ET collaboration efficiency in a dynamic environment. The excellence of the system is evaluated using various metrics, such as $\left\{(re_{utilization}\;),(w_{b}\;),\;(c_{q}\;),\;(R_{a}\;),\;(R_{c}\;and\;(R_{p})\right\}$. In MARL, $a_{j}$ adjusts their $ac_{j}$ to ensure the goal and balance the exploitation and explorations. During this process, the PPO learning process minimizes conflicts and improves the overall decision-making in task allocation. The iterative procedure reduces the computation difficulties and overall productivity in an ET dynamic environment. The reward mechanism clearly shows MARL’s efficiency while allocating tasks to the members according to their skills and priorities. The interaction between the team members and agents improves the overall collaboration in the ET environment. Frequently updating policies and rules helps maximize the overall team efficiency. Then, the collaboration efficiency is explored in terms of task completion time ($tc_{time}$), communication efficiency ($c_{effi}),\;conflict\;resolution\;speed\;(\;cf_{speed})$ and team cohesion score ($tc_{score})$. Then, the obtained collaboration efficiency is shown in Fig 6.

Fig 6 shows the collaboration efficiency analysis of the MARL framework in the ET environment. The collaboration efficiency is determined using $tc_{time}$ described in Fig 6a, which is evaluated in three different phases. From the analysis, the $tc_{time}$ value is higher in the initialization phase because of agent suboptimal coordination. After understanding correctly, the agent observes and learns the task features, allocation is performed effectively, and stability is maintained throughout task completion. The effective interaction and communication process reduce the completion time. The $c_{effi}$ Also, the minimum is at the beginning of the interaction because of unclear coordination and redundant interactions. The enhancement in understanding improves the overall $c_{effi}$ which indicates that the system ensures effective collaboration in the ET team process. Similarly, $\;cf_{speed}\;and\;tc_{score}$ value also having the minimum value in the initial stage; once the agent understands the overall complexity of task allocation, the entire collaboration efficiency is improved, as described in Fig 6c and 6d. These findings illustrate how MARL increases the level of cooperation by enhancing the division of agents, communication, the resolution of conflicts, and the unification of the team, which improves performance and productivity over time. The constant growth observed in all metrics reflects the system’s flexibility and demonstrates the opportunities MARL provides to enhance team dynamics. Then, the efficiency of this MARL study is evaluated using the respective case study analysis.

## 4. Research case analysis

This section examines how well the Multi-Agent Reinforcement Learning (MARL) framework analyses entrepreneurial team (ET) collaboration. Before assigning jobs, the analysis evaluates each agent’s behaviours, skills, and priorities. State, action, and reward reinforcement learning improves cooperation efficiency and business results iteratively. Python-based reinforcement learning programmes generated simulated datasets for the case studies, ensuring controlled and reproducible framework performance evaluation. No real-world or private data was used, hence no external data access or written agreements were needed. This method meets the journal’s ethical criteria because it doesn’t involve humans or confidential data. The hypotheses are explained in Table 2(a) and Table 2(b).

**Table 2:** (a) Hypothesis–reference mapping for MARL-based entrepreneurial team collaboration. (b) Various aspects of hypothesis exploration.

Hypothesis	Description	Supporting references	Rationale
**H1**	Task completion efficiency improves via optimized task allocation.	[15], [21,25,27]	Han [15] and Lv et al. [25] discuss skill-based member selection and DRL-based team formation; Du [21] and Zhou et al. [27] address scheduling and coordination using MARL.
**H2**	Communication efficiency improves by minimizing irrelevant interactions.	[14], [18,19,23]	Krawczyk-Bryłka [14] emphasizes effective ET communication; Covin [18] and Donbesuur [19] support goal alignment; Yang et al. [23] enhances communication via curriculum-based PPO.
**H3**	Conflict resolution time is reduced through adaptive strategies.	[16], [21,26]	Chughtai [16] covers adaptive leadership; Du [21] and Duraimutharasan [26] highlight learning-based collaboration and human-machine decision-making.
**H4**	Workload is balanced across team members to improve productivity.	[25], [27,28]	Lv [25] uses DRL for optimal team assignment; Zhou [27] and Tu [28] emphasize task distribution and adaptive role learning in multi-agent systems.
**H5**	Goal alignment and interaction enhance team cohesion.	[14], [17,18,20]	Bouncken [17] and Covin [18] analyze team synergy and cohesion; Sutrisno [20] links technology to goal achievement; [14] supports team dynamics education.
**H6**	Teams adapt to dynamic environments for sustained performance.	[16], [21,23,28]	Chughtai [16] on self-efficacy; Yang [23] and Tu [28] on adaptive learning; Du [21] for safe MARL in dynamic environments.
**H7**	MARL scales with team size while maintaining collaboration efficiency.	[22], [23,27]	Jeloka [22] introduces mean-field MARL for large teams; Zhou [27] and Yang [23] test scalability and PPO stability.
**H8**	Reward mechanisms guide agents toward aligned, efficient actions.	[23], [26,27]	Yang [23] defines PPO reward shaping; Duraimutharasan [26] and Zhou [27] use performance feedback and incentive-based learning.
**H9**	Learning phase significantly improves collaboration efficiency.	[21], [22,24,25]	Du [21] and Jeloka [22] show learning dynamics; Zheng [24] shows personalized growth; Lv [25] applies DRL learning in large teams.
**H10**	Stabilized performance achieved in long-term collaboration.	[20], [23,28]	Sutrisno [20] supports innovation over time; Yang [23] and Tu [28] emphasize long-term policy convergence in MARL.
***Hypothesis exploration***			
**(b) Various aspects of hypothesis exploration.**			
**Aspects**	**Hypothesis**	**Results**	
**Task completion** ${𝐭}_{𝐜𝐨𝐦}$	H1: The framework minimizes the ${\text{t}}_{\text{com}}$ time by fine-tuning the task allocation according to agents’ skills and reducing the redundancies in ET.	Confirmed: Task completion time decreased significantly due to optimized task assignments reducing overlap and delays.	
**Communication** $\;{𝐜}_{𝐨𝐩}$	H2: The MARL enhances ${\text{c}}_{\text{op}}$ by minimizing irrelevant and redundant interactions and ensuring effective communication between team members.	Supported: Communication became more efficient with fewer unnecessary interactions, improving overall information flow.	
**Conflict resolution** $𝐜{𝐟}_{𝐫𝐞}$	H3: The MARL quickens $\text{c}{\text{f}}_{\text{re}}$ in ET by permitting agents to understand the adaptive strategies for handling disagreements.	Validated: Conflict resolution time was shortened as agents learned adaptive strategies for managing disputes.	
**Workload balance** ${𝐰}_{𝐛}$	H4: MARL gives the ${\text{w}}_{\text{b}}$ environment among team members to reduce the differences in effort and task management	Confirmed: Workload distribution became more equitable, reducing strain on individual members and improving team productivity.	
**Team cohesion** ${𝐭}_{𝐜𝐨}$	H5: MARL-using entrepreneurial teams (ET) are more likely to achieve high team cohesion by aligning goals and improving interactions	Supported: Teams showed stronger cohesion, with better alignment and mutual support observed over time.	
**Adaptability** ${𝐑}_{𝐚}$	H6: Framework enhances the ${\text{R}}_{\text{a}}$ of members in the dynamic environment under various changing conditions.	Verified: Team members adapted more quickly to changing priorities and conditions, maintaining collaboration effectiveness.	
**Scalability** ${𝐑}_{𝐬}$	H7: The MARL scales effectively for large ET to enhance the collaboration efficiency	Confirmed: The framework maintained performance and collaboration quality even as team size increased.	
**Reward Optimization** ${𝐑}_{𝐨𝐩}$	H8: The MARL uses reward mechanisms to align the agent’s actions to enhance the goal and ET efficiency.	Validated: Reward allocation guided agents towards behavior that improved overall team efficiency and goal attainment.	
**Learning** ${𝐋}_{𝐩𝐡}$	H9: The framework significantly improved collaboration efficiency during the learning phase.	Supported: Collaboration metrics improved steadily as agents learned and adapted during initial deployment.	
**Stabilization – long short-term efficiency** $𝐒{𝐋}_{𝐞𝐟𝐟}$	H10: ET accomplishes the non-optimal collaboration excellence in the stabilization phase.	Observed: Teams reached a steady state of collaboration with consistent performance, though slight fluctuations remained.	

The data are taken from Academic and Entrepreneurial Development Dataset [30]. Undergraduates’ academic, behavioral, and entrepreneurial traits are recorded in this dataset in real-time. It includes 214,354 records with more than 40 variables, pulled from a variety of sources, including demographics, academic achievement, extracurricular activities, personality traits, and psychological characteristics. Created with educational and entrepreneurial research in mind, it yields useful information about talent prediction, skill development, and career outcomes. The analysis was conducted using Python 3.11, with TensorFlow and OpenAI Gym frameworks to implement the Multi-Agent Reinforcement Learning (MARL) model. Each agent represented an entrepreneurial team member, and the learning environment was designed to optimize communication, coordination, and decision-making outcomes. The MARL method was chosen due to its ability to model interdependent adaptive behaviors that characterize real-world entrepreneurial collaboration. Key hyperparameters were tuned through sensitivity analysis, including a learning rate of 0.001, a discount factor of 0.95, and an exploration decay rate ranging from 0.9 to 0.1 across 500 episodes. A reward convergence threshold of 10 ⁻ ³ was applied to ensure training stability. The experiments were repeated five times, and the averaged results were used to validate model reliability and consistency.

Table 2(a) presents a structured mapping between each research hypothesis (H1–H10) and the supporting scholarly references cited in the literature review. The studies of Han [15], Du [21], Lv [25], and Zhou [27] support H1 on task completion efficiency, while Krawczyk-Bryłka [14], Covin [18], Donbesuur [19], and Yang [23] support H2 on communication efficiency. Adaptive leadership, entrepreneurial team dynamics, DRL, MARL, incentive mechanisms, scalability, and long-term collaboration references support hypothesis H3–H10. It highlights how each hypothesis is grounded in previous research findings, ensuring theoretical relevance and academic rigor in the development of the MARL-based entrepreneurial team collaboration framework.

### 4.1 Case study discussions

The hypothesis from H1 to H10 explores the efficiency of the MARL framework in examining the entrepreneurial team’s collaboration efficiency (ET). Here, three case studies are utilized to investigate the efficiency of the MARL system, which is described as follows.

#### Research case 1: Collaborative product development.

A complete product lifecycle depends on the designer, engineer, and marketer’s work because the product should be designed with specific prototypes, and marketing should be created to improve overall efficiency. The entire project team works in a dynamic environment to meet the customer requirements. These scenarios address the $H\text{1}(t_{com})$, $H\text{4}(w_{b\;})$, and $H\text{5}\;(t_{co})$; along with this case study evaluated using the $t_{com}$ time at every stage, $w_{b\;}$among the team members and $t_{co}$ according to the goal alignment. For this case, the ET should be created for specific tasks with constraints defined in Table 3.

**Table 3:** Task and constraints for case 1.

Task	Design (*D*), Prototype (*P*) and Market (*M*)
Constraints	Limited resources, interdependent tasks and time-bound deliverables
Actions	Information sharing, resource allocation and goal prioritizing

According to the above task, constraints and actions, the reward $r_{j}$ is computed using the MARL framework to increase the $t_{co}$, $w_{b}$ and $t_{com}$. First, the task-finishing reward $R_{t}$ has to be allocated for every member $m_{j}$ because the $r_{j}$ helps to complete the task in a fast manner. For every $t_{i}$ the framework follows the {D, P and M} strategies to complete the $t_{j}$ at particular timeline T, and the system gives penalties for every delay. Then, the reward is defined as $R_{t}=\frac{\alpha }{T_{com}}$; the scaling factor is defined as α. The computed $R_{t}$ value is given to the agent $a_{j}$ depending on their performance and the team’s cumulative performance. The $a_{j}$ receives the high $R_{t}$ value according to the fastest completion. The $a_{j}\;$are act independently at the initial stage, which leads to lower $r_{j}$ value due to the improper communication. Once the framework is stable, the $a_{j}\;$receives the high $r_{j}$ value. The $R_{t}$ is given to the $a_{j}$ depending on the $w_{b}$ because the member should be balancing their work in a dynamic environment. The $R_{t}$ concerning $w_{b}\;$is computed as $R_{b}=\text{1}-\frac{\left|W_{i}-\overset{―}{W}\right|}{\text{max}\left(W\right)}$. If any team member has an imbalance in the workload, the entire performance is affected. At the same, E handles more than 50% of the task at the initial stage, which reduces the $R_{b}$ to 0.7, and the stabilization phase, the $R_{b}$ increases to 0.97. Then, $R_{t}$ in cohesion process $R_{c}$ motivates $a_{j}$ cooperations to achieve their goal in minimum time $\left(R_{c}=goal\;alignment\;score\right)$. The $R_{c}$ is obtained from communication quality, task synchronization and mutual support. Therefore, $c_{effi}\;and\;\text{min}\left(R_{cf}\right)$ improves the $R_{c}$. As same in the initial stage, $a_{j}$ obtained minimum $R_{c}=\text{0}.\text{4}$ value when in the stabilization face, the $R_{c}$ value increased up to 0.95. Then, the combine $R_{total}$ is defined in Equation (11)


$$\left\{ \;\;\;\begin{array}{c} R_{total}=w_{t}R_{t}+w_{b}R_{b}+W_{c}R_{c}\\ R_{t}=\frac{\alpha }{T_{com}}\\ R_{b}=\text{1}-\frac{\left|W_{i}-\overset{―}{W}\right|}{\text{max}(W)}\\ R_{c}=goal\;alignment\;score\\ w_{t},\;w_{b}\;and\;w_{c}\;is\;weight\;values\; \end{array}\right.\;$$ (11)

In the Equation (11), $w_{t}$ is a higher value at the initial stage to prioritize the $t_{effi}$ and $w_{b}and\;w_{c}$ is augmented gradually to boost cohesion and balance. According to the discussions, the $R_{t}$ Case 1 is illustrated in Table 4.

**Table 4:** $R_{t}$ Mechanism for case 1.

	Initi_phase_	Learn_phase_	stab_phase_
${{𝐓}_{𝐜𝐨𝐦}}_{𝐭}$	High	Improves their process	Drops
${𝐖}_{𝐛}$	Evident	Reduce to 20%	Reduce to an optimal level
${𝐓}_{𝐜𝐨𝐡\;}$	Low	Improve score to 0.65	Peat at 0.95
Reason	${\text{a}}_{\text{j}}$ prioritize independent goal	${\text{a}}_{\text{j}}$ optimizing ${\text{t}}_{\text{alloc}}$ and learning interactions	Stable policies and manage the continuous collaboration

From Table 4 it clearly shows that for case 1, the product development process improves their performance from $initi_{phase}$ to $stab_{phase}$ which means the effective computation of MARL identifies the $r_{j}$ for every $t_{com}$, $w_{b}$ and $t_{coh}$ to ensure stable policies and maintain continuous collaborations. Then, case 1 is evaluated using the $t_{com}$, $w_{b}$ and $t_{coh}$ metrics with three stages $initi_{phase}$, $learn_{phase}\;and$ $stab_{phase}$ at different episodes, and the results obtained are shown in Table 5.

**Table 5:** Efficiency analysis of case 1.

	Initi_phase_	Learn_phase_	stab_phase_
${{𝐓}_{𝐜𝐨𝐦}}_{𝐭}$	0.4	0.7	0.967
${𝐖}_{𝐛}$	0.5	0.84	0.97
${𝐓}_{𝐜𝐨𝐡\;}$	0.43	0.65	0.95

Table 5 illustrates that the efficiency analysis of case 1 with three metrics $t_{com}$, $w_{b}$ and $t_{coh}$. The analysis clearly says that the $t_{com}$ gradually increased from $initi_{phase}$ to $stab_{phase}$ because of the effective coordination that validates the hypothesis H1. Then $w_{b}$ evenly distributed in three phases, which supports the hypothesis H4 and $t_{coh}$ helps $a_{j}$ learn their strategies to meet the goal and enhance the cohesion, which satisfies hypothesis H5. Therefore, this case study clearly shows that the MARL approach effectively balances the agent’s roles, optimizes collaborations and improves the ET to fulfil the objectives successfully.

#### Research case 2: Start-up crisis management.

Every start-up team and business faces a crisis because of fast task reallocation, resource shortages and resolution conflicts between the members with high responsibilities. This case study helps to address the conflict resolution $\left(cf_{res}\right)$(H3), communication optimization $\left(c_{op}\right)$ (H2) and adaptability $\left(r_{a}\right)$ (H6) hypothesis. These hypotheses are handled with the help of different metrics such as conflict resolution time $\left(cf_{res_{t}}\right)$, communication efficiency $\left(c_{effi}\right)$ and adaptability score according to $a_{j}$ $\left({R_{a}}_{score}\right)$. This case study uses three agents: a developer (D), a tester (T) and a product manager (PM). The agents are represented as $a_{j}=\{D,T,\;PM\}$; their objective is to improve the start-up productivity by reducing the conflicts delay and aligning the ET goal. During the every task allocation and process, $r_{j}$ is utilized for creating effective software development. First, $t_{com}$ bonus value is given for every $a_{j}$ of team members $M_{j}$ to motivate to complete the task $t_{j}$ at a given time. Then the $r_{j}$ for $t_{com}$ is defined as $R_{t}=\beta_{\text{1}}.\left(T_{target}-T_{actual}\right)$ in which expected ($T_{target})$ and $T_{actual}$ actual completion time for $t_{j}$ is estimated along with the time weight value $\beta_{\text{1}}$. Then rewards $r_{j}$ is allocated to the $a_{j}or\;M_{j}$ for successful address of bugs, which is defined as $R_{bug}=\beta_{\text{2}}.\left(\text{1}-\frac{Open_{bug}}{total_{bug}}\right)$. The bug-resolving characteristics help to understand the $a_{j}\;or\;M_{j}$ skill for particular software development. In addition, the collaboration score $R_{coll_{score}}$ value is provided for $a_{j}\;or\;M_{j}$ to successive prioritize tasks and communication. The $R_{coll_{score}}=\beta_{\text{3}}.\;coll_{index}$. Finally, the penalty $\left(P_{t}\right)$ also given to the $a_{j}\;or\;M_{j}$ for their negative actions or idle state in $t_{alloc}.$ Therefore, the entire $R_{total}$ value is estimated using Equation (12).


$$\left\{ \;\;\;\begin{array}{c} R_{total}=R_{t}+R_{bug}+R_{coll_{score}}+{\text{P}}_{\text{t}}\\ R_{t}=\beta_{\text{1}}.\left(T_{target}-T_{actual}\right)\\ R_{bug}=\beta_{\text{2}}.\left(\text{1}-\frac{Open_{bug}}{total_{bug}}\right)\\ R_{coll_{score}}=\beta_{\text{3}}.\;coll_{index}\;\\ P_{t}=-\beta_{\text{4}}\\ \beta_{\text{1}},\;\beta_{\text{2}},\;\beta_{\text{3}}\;and\;\beta_{\text{4}}\;is\;weight\;values\; \end{array}\right.\;$$ (12)

These rewards are obtained from the MARL process in which the system inputs the backlog size, task progress, communication frequency, and bug severity. The inputs are processed by $sh_{net}$ to derive the features fed into the policy and value network to get the optimized actions and respective values for each pair. During the $ac_{j}$, goal reprioritization, bug escalation, and task allocation process is performed. According to these actions, the PPO algorithm updates the policies to improve the overall collaboration goal. Based on the discussion, the obtained values for case 2 are shown in Table 6.

**Table 6:** Efficiency analysis of case 2.

	Initi_phase_	Learn_phase_	stab_phase_
${T_{com}}_{t}$	17days	13 days	6 days
$bug_{res}$	45%	80%	95%
$colla_{qual}$	0.43	0.67	0.97
$It_{per}$	34%	12%	3%

Table 6 illustrates the efficiency analysis of case 2, which is determined in terms of task completion time ($T_{com_{t}})$, bug resolution $\left(bug_{res}\right)$, idle time percentage $\left(it_{per}\right)$and collaboration quality $\left(col{la}_{qual}\right)$. From the analysis, $T_{com_{t}}$ value is minimized from $initi_{phase}$ to $stab_{phase}$ which indicates that the $a_{j}$ manages the task allocation and prioritization time once they have the proper understanding of the task $t_{j}$. Then, for case 2, the $bug_{res}$ is increased by 45% from $initi_{phase}$ to $stab_{phase}$ (95%) that represented that, $a_{j}$ understand the bug type and prioritize according to their importance, augmenting the debugging time. The $colla_{qual}$ value is increased from 0.43 to 0.97, showing that MARL successfully improves teamwork, effective communication and understanding of ET goals. Finally, the $it_{per}$ is minimized from 34% to 3%, which shows that idle $a_{j}$ engaged in auxiliary roles and augmenting resource utilization. From the analysis, the mapping of the hypothesis concerning these metrics is shown in Table 7.

**Table 7:** Hypotheses mapping for case 2.

Metrics	Hypothesis
${{𝐓}_{𝐜𝐨𝐦}}_{𝐭}$	**H2: improves adaptability and optimizes task allocation.**
$𝐛𝐮{𝐠}_{𝐫𝐞𝐬}$	**H3: enhance cohesion and communication, which leads to optimized bug fixes.**
$𝐜𝐨𝐥𝐥{𝐚}_{𝐪𝐮𝐚𝐥}$	**H3: improves priorities alignment and communication to enhance the ET collaboration efficiency.**
$𝐈{𝐭}_{𝐩𝐞𝐫}$	**H6: minimize idle periods and balance resource optimization and workload.**

Case Study 2 is concerned with applying the MARL framework within an agile software development team. It attempts to address task reallocation, resource shortages and resolution of conflict issues. Results suggest that there were significant improvements across all metrics, and agents were able to learn how to coordinate themselves optimally, set shared objectives, and reduce idle time. The findings support the hypotheses H2 ($r_{a})$, H3 $\left(colla_{effi}\right)$ and H6 ${(res}_{utili})$ respectively.

#### Research case 3: Strengthening team for crowdfunding success.

The entrepreneurial side of the team continues to expand as they run a crowdfunding activity, growing from three to six agents with different roles: content creators, outreach managers and financial analysts in the company. This case study was used to address the scalability (sc)(H7), reward optimization $\left(R_{op}\right)$ (H8) and long-term and stabilization efficiency $\left(LS_{effi}\right)$ (H10). These hypotheses are evaluated using different metrics like collaboration efficiency $\left(colla_{effi}\right)$, total reward efficiency $\left(Ro_{effi}\right)$ and long-term stabilization $\left(Ls_{effi}\right)$. The case study uses the three $a_{j}$ such as product manager (PM), quality analyst (QA) and logistics coordinator (LC); $a_{j}=\left\{PM,\;QA\;and\;LC\right\}$. For every function, reward $r_{j}$ is tailored to improve overall manufacturing. The total reward $\left(R_{tot}\right)$ estimation for this case study 3 is shown in Equation (13).


$$\left\{ \;\;\;\begin{array}{c} R_{tot}={R_{pr}}_{t}+R_{dr}+R_{l}+R_{ci}+{\text{P}}_{\text{t}}\\ {R_{pr}}_{t}=\beta_{\text{1}}.\frac{units\;produced}{target\;unit}\\ R_{dr}=\beta_{\text{2}}.\left(\text{1}-\frac{defective\;units}{total\;units}\right)\\ R_{l}=\beta_{\text{3}}.\left(\text{1}-\frac{delivery\;delays}{total\;deliveries}\right)\\ R_{ci}=\beta_{\text{4}}.\;colla_{qu_{sc}}\\ P_{t}=-\beta_{\text{5}}\\ \beta_{\text{1}},\;\beta_{\text{2}},\;\beta_{\text{3}}\;and\;\beta_{\text{4}}\;is\;weight\;values\; \end{array}\right.\;$$ (13)

Initially, the production throughput $pr_{t}$ related rewards are computed as ${R_{pr}}_{t}=\beta_{\text{1}}.\frac{units\;produced}{target\;unit}$. After allocating the ${R_{pr}}_{t}$, the defects are examined and the $a_{j}$ or $M_{j}$ correctly identifies the defects, then defect reduction $R_{dr}$ is given as $R_{dr}=\beta_{\text{2}}.\left(\text{1}-\frac{defective\;units}{total\;units}\right)$. Along with this, logistic efficiency is evaluated, and the respective rewards are provided, which is defined as $R_{l}=\beta_{\text{3}}.\left(\text{1}-\frac{delivery\;delays}{total\;deliveries}\right)$. According to their performance, collaboration incentives $R_{ci}$ is given that is computed as $R_{ci}=\beta_{\text{4}}.\;colla_{qu_{sc}}$ and the penalty is also given for the downtime that is measured as $P_{t}=-\beta_{\text{5}}.\;D_{dura}$. Based on the discussion, the obtained values for case 3 are shown in Table 8.

**Table 8:** Efficiency analysis of case 3.

	Initi_phase_	Learn_phase_	stab_phase_
${R_{pr}}_{t}$	73%	88%	98%
$R_{dr}$	17%	9%	3.5%
$R_{l}$	65%	87%	97%
$R_{ci}$	0.55	0.85	0.96
$P_{t}$	23%	8.5%	2.7%

Table 8 clearly shows that case 3, based on MARL efficiency analysis, in which the ${R_{pr}}_{t}$ is increased from 73% to 98% of $initi_{phase}$ to $stab_{phase}$. The ${R_{pr}}_{t}$ increase shows that better task scheduling and coordination between the $m_{j}\;or\;a_{j}$. Then the $R_{dr}$ is reduced significantly from 17% to 3%, which creates an impact on QA and quality check prioritization. The $R_{l}$ is increased from 65% to 97% of $initi_{phase}$ to $stab_{phase}$ that represented supportive supply chain management. The $R_{ci}$ value is slightly improved from 0.55 to 0.96, effectively validating the team collaboration goal. finally, the $D_{dura}$ is reduced from 23% to 2.7%, showing that the project uses the optimized resources and minimum idle time. The analysis shows the mapping of the hypothesis concerning these metrics in Table 9.

**Table 9:** Hypotheses mapping for case 3.

Metrics	Hypothesis
${{𝐑}_{𝐩𝐫}}_{𝐭}$	**H7: improves operational efficiency**
${𝐑}_{𝐝𝐫}$	**H10: enhance adaptability and reduce the errors**
${𝐑}_{𝐥}$	**H8: optimize supply chain operations and minimize the bottleneck and delay**

Table 9 clearly shows that the MARL approach successfully addresses the scalability (sc)(H7), reward optimization $\left(R_{op}\right)$ (H8) and long-term and stabilization efficiency $\left(LS_{effi}\right)$. The results indicate the computed metrics, such as ${R_{pr}}_{t}$*,* $R_{dr}\;and\;R_{l}$ create an impact on the collaborative and adaptability capabilities of the system. Therefore, the research applies a multi-agent reinforcement learning (MARL) framework to improve the collaboration of entrepreneurial teams. The framework integrates team dynamics issues within varied case studies by improving task distributions, inter-team communication, and system usage proficiency. The results confirm that the framework works with each case, registering increased productivity, better utilization of resources, and higher quality of output, as measured against some standards. The insights suggest that MARL could improve the structure of team-based operations and improve their efficiency.

## 5. Conclusion

This study details a Multi-Agent Reinforcement Learning (MARL) framework to increase entrepreneurial team collaboration. The framework improves communication, resource utilisation, and downtime by continuously assessing team members’ competencies and dynamically assigning tasks. Individual performance rewards ease job allocation in dynamic circumstances and foster team cooperation. Agents’ role adaptation and alignment with shared goals promote productivity, lessen interpersonal disputes, and improve change adaptability. The framework was proven effective in various team-based situations through case studies in agile software development, manufacturing optimisation, and logistics coordination. Future research should integrate advanced AI models like deep neural networks to improve decision-making precision and scale the system to handle bigger, more varied teams. MARL’s effects on team performance, flexibility, and long-term company success must be assessed in entrepreneurial ecosystems in real time.The study uses simulated data, which may not accurately depict entrepreneurial teams. The MARL framework may be outdated, and scalability in bigger, heterogeneous teams is unknown. Agent modelling simplifies complex human behaviours, but real-time deployment in practice is still unexplored.
